# Estimation of prokaryotic supergenome size and composition from gene frequency distributions

**DOI:** 10.1186/1471-2164-15-S6-S14

**Published:** 2014-10-17

**Authors:** Alexander E Lobkovsky, Yuri I Wolf, Eugene V Koonin

**Affiliations:** 1 National Institutes of Health, National Center for Biotechnology Information, 8600 Rockville Pike, Bethesda, MD, 20892 USA

**Keywords:** supergenome, genome evolution, gene frequency distribution, ancestral reconstruction

## Abstract

**Background:**

Because prokaryotic genomes experience a rapid flux of genes, selection may act at a higher level than an individual genome. We explore a quantitative model of the distributed genome whereby groups of genomes evolve by acquiring genes from a fixed reservoir which we denote as supergenome. Previous attempts to understand the nature of the supergenome treated genomes as random, independent collections of genes and assumed that the supergenome consists of a small number of homogeneous sub-reservoirs. Here we explore the consequences of relaxing both assumptions.

**Results:**

We surveyed several methods for estimating the size and composition of the supergenome. The methods assumed that genomes were either random, independent samples of the supergenome or that they evolved from a common ancestor along a known tree via stochastic sampling from the reservoir. The reservoir was assumed to be either a collection of homogeneous sub-reservoirs or alternatively composed of genes with Gamma distributed gain probabilities. Empirical gene frequencies were used to either compute the likelihood of the data directly or first to reconstruct the history of gene gains and then compute the likelihood of the reconstructed numbers of gains.

**Conclusions:**

Supergenome size estimates using the empirical gene frequencies directly are not robust with respect to the choice of the model. By contrast, using the gene frequencies and the phylogenetic tree to reconstruct multiple gene gains produces reliable estimates of the supergenome size and indicates that a homogeneous supergenome is more consistent with the data than a supergenome with Gamma distributed gain probabilities.

## Background

The advances of comparative genomics have made it clear that gene exchange is the primary mode of evolution in prokaryotes [1-5]. The intensity of this exchange is such that several distinct gene families are lost and gained on average in a time it takes for a single amino acid substitution to be fixed in a conserved gene [6]. Because new genes predominantly originate by horizontal gene transfer (HGT) [7-9], it seems plausible that the unit of evolution in prokaryotes is not the genome but the supergenome, i.e. the collection of all genes available for HGT [10]. The rationale behind this way of thinking is that a novel gene discovered in one population can be acquired by another population. Although many distinct new genes arrive regularly, which of these genes are fixed varies greatly between populations [11,12]. Therefore, given a group of closely related prokaryotes, it is useful to know the size and composition of the pool of genes that can, in principle, be adopted by the organisms in the group. We denote this gene pool supergenome to differentiate it from the more common term pan-genome which we reserve for denoting the set of distinct genes empirically shown to be present in the chosen group. Note that the supergenome has been alternatively denoted as supragenome in [13].

In the years since the importance of HGT and distributed genomes in prokaryotes has been recognized, there has been considerable interest in estimating the supergenome size for diverse prokaryotes. The supergenome of a microbial species group cannot be characterized directly and can be estimated only from the analysis of samples of the relevant genomes using an explicit model of genome evolution. Such estimates have pointed to vast supergenomes for most prokaryotes because for the majority of prokaryotes, sequencing and analysis of new isolates did not show any signs of saturation of new gene discovery, suggestive of "open" supergenomes. However, for several bacterial species, new genomes add few new genes, indicating that these organisms have relatively small supergenomes [2,14,15]. Snipen and coworkers applied a binomial mixture approach to approximate the gene frequency distribution in an analyzed set of genomes and used it to estimate the supergenome size for a variety of bacteria [16,17]. Unlike the earlier attempts, this approach yielded closed, relatively small supergenomes that were only several fold larger than a typical genome in the group. Recently, the Infinitely Many Genes model of microbial genome evolution by gene replacement has been developed, under which the replacing genes are drawn from a formally infinite reservoir [18,19]. Estimates under this model have also suggested a closed supergenome for the cyanobacterium *Prochlorococcus*. However, the estimated supergenome was much larger than that predicted by the binomial mixture method. Given the discrepancies between the supergenomes estimated with different approaches, the validity of the underlying models of genome evolution and characteristic supergenome size for different prokarytoes remain open questions. This uncertainty motivated us to perform a systematic study of the supergenomes using a broad variety of approaches.

Here we describe and compare several methods for inferring the supergenome size and composition. We apply these methods to the task of estimating supergenome sizes of several groups of bacteria and discuss the robustness and accuracy of the results.

We find that supergenome size estimates based directly on the empirical gene frequencies are unreliable because they depend critically on the choice of the distribution of the gene gain probabilities in the supergenome. However, when the gene frequencies were used together with a phylogenetic tree to reconstruct the history of gene gain events, the numbers of multiple (repeated) gains could be used to obtain reliable estimates of the supergenome size. We further find that the homogeneous supergenome fits the reconstructed gains better than a supergenome in which gain probabilities are Gamma distributed.

## Methods

The supergenome is defined only in the context of evolution. Therefore the estimates of supergenome size must involve an explicit or implicit model of the evolution of gene content. Because only the extant genomes are available, all methods must address the problem of ancestral reconstruction. The methods that assume that genomes are random, independent collections of genes drawn from a supergenome (i.e. methods A, B, C and E below) imply that gene content evolution is so fast that every pair of genomes in the group has diverged as far as possible from each other in terms of their gene content, i.e. has reached the steady state of the stochastic process of gene acquisition.

For each method, the starting point is the assignment of homology relations among the genes of the chosen group of genomes. We omit the details of this process here [20] and only indicate that genes are clustered into families using the BLAST search hits with scores and random expectation values above an appropriate threshold of significance [21]. The outcome of this homology identification procedure is a list of families and an assignment of each gene to a unique family. Genomes can contain multiple representatives of a family (paralogs).

### A: Pan-genome growth curve

Given *G *genomes, method A, computes the pan-genomes, i.e. the number of distinct genes found in a group of genomes, for of all groups of size *g ≤ G *and plots the average pan-genome size *P*(*g*) among these groups as a function of *g*. This so called pan-genome growth curve or its derivative, i.e. the average number of new genes discovered when (*g *+ 1)*^st ^*genome is added to a group of *g *genomes, is then fit by either an exponential [22] or a power law function [14]. Conclusions about the size of the supergenome are made depending on the existence of the asymptote or the value of the exponent of the power law. The power law (but not the exponential) functional form of the pan-genome growth curve has a theoretical basis. When words are drawn at random from a vocabulary in which word frequencies are power law distributed (Mandelbrot), the number of distinct words discovered after *K *samples grows initially as a power law and asymptotes to the vocabulary size when *K *is sufficiently large [23]. However, when applied, the results of the power law fits are frequently outside of the validity range of the Mandelbrot/Heaps construction [14]. When the extracted exponent is indeed within the range of validity of Heaps law, the supergenome size can be estimated by examining the *deviation *from the power law. To our knowledge, analysis of this deviation so far has not been reported. It is likely that the sets of genomes analyzed with this technique have not been large enough to exhibit a reliably detectable deviation from the power law growth of the pan-genome. We therefore did not pursue the application of this method in this work.

### B: Binomial mixtures

The binomial mixtures method (B) also assumes that genomes are random, independent collections of genes drawn from a heterogeneous supergenome which consists of several distinct categories. Each category is characterized by a detection probability (probability that genes from this category are present in a genome) and admixture fraction which is the portion of the genome allocated to this category [13,16,17,24]. The detection probabilities and the admixture fractions are estimated with a maximum likelihood method. The empirical data used for the ML estimates are the numbers *γ_g _*of families found in exactly *g *out of *G *genomes for each 1 *≤ g ≤ G*. One of the categories is considered indispensable, i.e. it has a unit detection probability. If there are *k *categories, the model has 2*k − *2 adjustable parameters [16]

The final ingredient in this approach is the selection of the optimal number of categories to minimize the Bayesian Information Criterion (*BIC*) in order to avoid over-fitting.

### C: Capture-recapture

The task of estimating the number of genes that have not yet been observed after a certain number of samples (genomes) are taken is akin to the population size estimate problem in experimental ecology [25]. If all genes are equally likely to be "captured," the classic capture-recapture formula gives the most likely number of genes not yet captured as $\gamma_{1}^{2}\text{/}\gamma_{2}$ where *γ_g _*is defined in the previous subsection [26]. The capture-recapture method is a version of the binomial mixture method with a homogeneous reservoir. Because only a small fraction of empirical information about gene frequencies is utilized in the estimate, the capture-recapture method is used here only as a reference.

### D: Gamma distributed gain probabilities

The binomial mixtures model (B) assumes that genes arrive from a small number of homogeneous reservoirs, i.e. within each reservoir genes have the same probability of being transferred into the genome. The empirical data seem to indicate that the distribution of gain probabilities is broad as reflected in the moderate optimal number of reservoir types *k *which minimizes the *BIC*. It is of interest therefore to ascertain the effect of a model in which gain probabilities are derived from a parametrized distribution on supergenome estimates. We aim to determine whether the gene frequencies themselves contain enough information about the supergenome so that estimates of its size are robust with respect to model selection.

Consider a supergenome of size *S *in which every gene *i *has a distinct gain probability *p_i_*. Assemble *G *genomes of size *M *by making random independent samples from the supergenome. The probability *Q_i_* that gene *i *is present in a single genome is

(1) $$Q_{i}=1-{\left(1-p_{i}\right)}^{M},$$

and therefore the probability $R_{i}^{g}$ that gene *i *is present in exactly *g *out of *G *genomes is

(2) $$R_{i}^{g}=\left(\begin{array}{c} G\\ g \end{array}\right)Q_{i}^{g}{\left(1-Q_{i}\right)}^{G-g}.$$

To compute the likelihood of a set of empirically observed *γ_g_*'s given the model, we need to compute the probability *θ_g _*that a randomly chosen family from the supergenome is found in *g *genomes

(3) $$\theta_{g}=\frac{1}{S}\overset{S}{\underset{i=1}{\sum }}R_{i}^{g}$$

Further progress is made by assuming that the gain probabilities *p_i _*are derived from a gain rate probability distribution *P*(*r*) whereby *S *rates *r_i _*are drawn from the *P*(*r*) and the gain probabilities are constructed via a normalization $p_{i}=r_{i}/\sum_{j}r_{j}$. The probability distribution *P*(*r*) is parametrized and the optimal parameters are obtained via the maximization of the zero-truncated log-likelihood introduced by Snipen *et al *[16]

(4) $$l=\overset{G}{\underset{g=1}{\sum }}\gamma_{g}\text{log}\frac{\theta_{g}}{1-\theta_{0}}.$$

The likelihood expression above is approximate because it assumes that the empirically observed gene counts *γ_g _*are independent of each other. Because computing the likelihood directly is computationally infeasible, an ad-hoc approximate expression must be used. Although the full exploration of the properties of the likelihood expression proposed by Snipen *et al *[16] is beyond the scope of this manuscript, we performed a limited test of the goodness of the approximation under the conditions which allow the direct computation of the likelihood.

**Figure 1:**
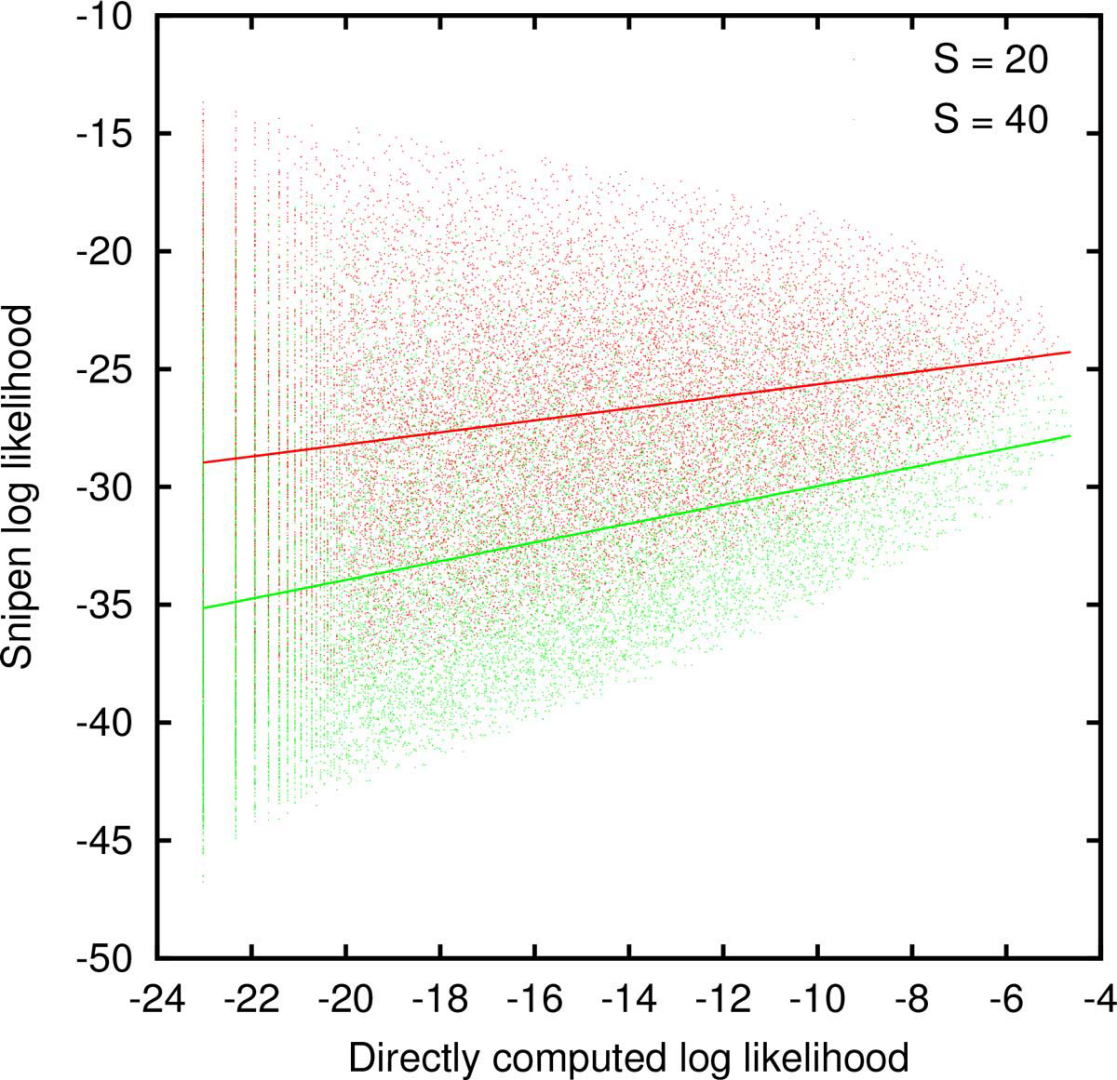
**The approximate log likelihood introduced by Snipen *et al *for every possible set of gene counts *γ_g_*vs**. the directly computed log likelihood for *G *= 5 and *M *= 10. The solid lines are linear fits to the *S *= 20 (red) and *S *= 40 (green) clouds.

When the number of genomes *G*, the genome size *M *and the reservoir size *S *are small, the likelihood can be computed directly by repeatedly constructing the genomes, computing *γ_g_*, and keeping track of the number of occurrences of each distinct set of *γ_g_*'s. If, at the same time, the probabilities *θ_g _*are computed, the Snipen likelihood can be evaluated for each set of *γ_g_*'s. Figure 1 confirms that the Snipen log likelihood positively correlates with the directly computed log likelihood when the reservoir size *S *is fixed although the scatter is large. Because we ultimately seek to estimate the reservoir size *S *by maximizing likelihood, it is more relevant whether the ad-hoc likelihood tracks the directly computed likelihood for a fixed set of *γ_g_*'s. To address this question, we fixed *G *= 5 and *M *= 10 and computed the direct and ad-hoc likelihoods, for all possible sets of *γ_g_*'s with reservoir sizes *S *= 10, 15, 20, 25, 30, 40, 45, and 50. Then for each set of *γ_g_*'s that occurred for at least 4 different values of *S*, we computed the linear correlation coefficient between the directly computed and the ad-hoc likelihoods. Among over 25,000 distinct sets of *γ_g_*'s the average Pearson correlation coefficient was 0.6 with a standard deviation of 0.5. Therefore, at least on average, minimizing the ad-hoc likelihood would yield a similar value of *S*. It is plausible that the degree of interdependence between the gene counts *γ_g _*is reduced when the genome size and the reservoir size are large, further narrowing the difference between the directly computed and the approximate likelihoods. We therefore proceed with exploring the consequences of maximizing the Snipen zero-truncated likelihood in the remainder of this work.

Computation of *θ_g_*'s is substantially simplified by taking *S *and *M *to infinity simultaneously while keeping their ratio *S/M *= *s *constant. We obtain

(5) $$\theta_{g}=\left(\begin{array}{c} G\\ g \end{array}\right)\int_{0}^{\infty }dr\;P\left(r\right){\left(1-e^{-r\text{/}s\langle r\rangle }\right)}^{g}e^{-\left(G-g\right)r\text{/}s\langle r\rangle },$$

where $\left\langle r\right\rangle =\int_{0}^{\infty }rP\left(r\right)\;dr$ is the mean gain rate which can be eliminated from Eq. (5) by a change of variables because the probabilities sum to unity $\sum_{i}\;p_{i}=1$. Thus, we need only consider distributions *P*(*r*) with unit mean because relative gain probabilities are independent of .

Here we let *P*(*r*) be a Gamma distribution of shape parameter *α *and scale parameter 1*/α*. When *α *is large, the distribution is peaked and most families are almost equally likely to be gained. When *α *is small, gain probabilities are broadly distributed. Note that the shape parameter *α *and relative supergenome size *s *= *S/M *are the only parameters in this model.

### E: Stochastic genome evolution on a tree

The supergenome estimation methods A, B, C and D discussed above assume that genomes are random and independent samples from a structured pool. In reality genomes evolve from a common ancestor and therefore can be substantially different from being random and independent. If the random, independent assumption is to be relaxed, quantitative information about the degree of divergence for every pair of genomes must be utilized. This information is contained in the phylogenetic tree. We base our calculations on the tree from the Microbes Online resource which is constructed using the concatenated sequences of multiple conserved genes [27]. Because this tree reflects evolution of nucleotide sequences rather than evolution of gene content, it adds information that is not taken into account in approaches A, B, C and D.

We sought to extend model D to include explicit genome evolution on the known phylogenetic tree. To this end, we assume that genes are lost with a certain rate *R *per unit time measured in nucleotide substitutions per site. When a gene is lost, its replacement is drawn from the supergenome according to probabilities *p_i_*. The simulation of this process is explicit: *S *numbers are drawn from *P*(*r*) and normalized to obtained the set of gain probabilities. The root node's genome of size *M *is drawn from the supergenome according to the gain probabilities. Genomes are evolved along the tree via the stochastic simulation of the gene replacement and the gene frequencies of the leaf nodes are computed. The estimates of the model *θ_g_*'s are obtained by averaging the simulated gene frequencies over 10^4 ^instances of the simulation.

The three parameters *α, S/M *and *R *are obtained by maximizing the likelihood in Eq. (4) for a fixed *M *= 500. Model E reduces to model D in the limit of infinite loss rate *R*.

### F: Maximum likelihood reconstruction of multiple gains

Instead of simulating losses and gains to estimate gene frequencies, one can alternatively estimate the numbers of gains and losses by reconstructing the gene content of ancestors from the gene content of the extant genomes. To this end, we use the Count method [28] which assumes that the copy number of each family behaves according to a birth-death model which includes gains, losses and duplications. Count uses maximum likelihood to optimize the parameters of the model given the topology of the phylogenetic tree and the gene content of the leaf nodes of the tree. Another important consideration is the number of categories of the Gamma distributed parameters of the underlying birth-death model. As explained below, we used 4 categories for loss, transfer and duplication rates giving 64 total categories for gene families.

Once the parameters of the birth-death model are estimated, Count computes *a posteriori *probabilities of gains, losses, expansion, and contraction for every family on every branch of the tree. Our goal is to identify all gene acquisitions and use the their number to estimate the size of the pool from which genes originate. An acquisition can result in a gain of a gene family or expansion of a pre-existing family. Existing families can expand by duplications as well as by acquisitions although it is unfeasible to quantitatively disentangle duplications from acquisitions [28]. We therefore use only gains (acquisitions of absent families) as the lower bound on the number of gene acquisitions. The upper bound on the number of acquisitions can be obtained by assuming that the duplications can be neglected and all gains and expansions are due to acquisitions. The lower bound on the number acquisitions translates into an upper bound for the supergenome size estimate and *vice versa*.

For each family, we compute the Count-estimated number of acquisitions (either only gains or gains plus expansions) over the entire tree. Let *P *be the number of families acquired at least once and *K *be the total number of acquisitions which can be broken down further into the numbers *n_k _*of families acquired *k *times so that $\sum_{k=1}^{K}n_{k}=K$.

To estimate the supergenome size *S*, we use two alternative models of the reservoir from which the genes are acquired. Model F1 assumes that all families have an equal chance of being drawn from the reservoir. The likelihood of drawing *P *distinct families in *K ≥ P *attempts is

(6) $$L=\frac{\left(\begin{array}{c} S\\ P \end{array}\right)P!P^{K-P}}{S^{K}},$$

The likelihood is maximized when

(7) $$\frac{\partial \text{ln}L}{\partial S}\approx \text{ln}\frac{S}{S-P}-\frac{K}{S}-\frac{P}{2S\left(S-P\right)}=0,$$

where we use the Stirling's approximation for the factorials log *n*! *≈ *(*n*+1*/*2) log *n−n*. Thus, given the total number of acquisitions *K *and the number of families *P *acquired at least once, we estimate *S *by solving Eq. (7). In addition, assuming that the likelihood function can be approximated by a Gaussian near its maximum, we can compute the 95% confidence interval Δ*S *for the estimate of *S *via

(8) $$\Delta S=\frac{2}{\sqrt{\frac{1}{S-P}-\frac{1}{S}+\frac{K}{S^{2}}+\frac{1}{2S^{2}}-\frac{1}{2{\left(S-P\right)}^{2}}}}$$

Model F2 allows the probabilities *p_i _*of receiving gene *i *from the supergenome to vary. To connect with models D and E, we assume that the gain probabilities are derived from a Gamma distribution with the shape parameter *α*. The probability *λ_k _*that a randomly picked reservoir gene was sampled *k *out of *K *times is

(9) $$\lambda_{k}=\frac{1}{S}\overset{S}{\underset{i=1}{\sum }}\left(\begin{array}{c} K\\ k \end{array}\right)p_{i}^{k}{\left(1-p_{i}\right)}^{K-k}$$

The model parameters are obtained by maximizing the zero-truncated log-likelihood of observing the numbers *n_k _*families acquired *k *times

(10) $$l=\overset{K}{\underset{k=1}{\sum }}n_{k}\text{log}\frac{\lambda_{k}}{1-\lambda_{0}}.$$

In this case, the uniform gain probability model, in which *p_i _*= 1*/S*, is recovered in the *α → ∞ *limit. To ascertain whether non-uniformity of the gain probability is supported by the data, we compare the Bayesian Information Criterion BIC = *−*2*l − c *log *P*, where the number of parameters is *c *= 1 for the uniform and *c *= 2 for the Gamma distributed gain probability models.

## Empirical data sets

Estimation of the size and composition of the supergenome requires selecting groups of high quality genomes, establishing homology relationships among their genes and building a phylogenetic tree reflecting the evolutionary distances between the genomes. We selected 9 groups of 10 bacteria genomes from the family *Enterobacteriacae *(see the Additional file 1 for details) using the Microbes Online resource which provides the tree as well as the complete genomes. The selected groups consist of closely related organisms whose genome sizes differ by at most 5%. The groups share the same common ancestor. The rationale is to examine the variability in supergenome estimates for groups of organisms of roughly the same diversity and having the same ancestor. Average genome size, root to tip distance and family copy numbers are summarized in Table 1.

**Table 1:** Groups used in testing the supergenome size estimation.

Grp.	Gen. size	Br. len	Cpy num.	B: *S*	B: *BIC*	C: *S*	D: *BIC*	E: *BIC*
1	4132	0.019	1.53	4,381	15,576	5,348	15,565	15,569
2	4275	0.075	1.58	8,206	21,445	14,347	22,236	22,235
3	4363	0.074	1.57	13,767	24,930	31,086	27,765	26,752
4	4444	0.043	1.56	9,823	25,017	23,448	25,452	25,459
5	4576	0.034	1.56	8,905	24,041	16,746	24,464	24,473
6	4721	0.021	1.56	8,974	22,932	13,918	23,040	23,050
7	4920	0.018	1.54	13,292	26,048	29,819	28,736	28,381
8	5123	0.029	1.66	9,172	26,095	14,703	27,135	27,145
9	5336	0.029	1.63	13,158	26,036	27,974	27,454	26,731

Homology relationships are established by performing a BLAST search of all against all proteins in each group separately and clustering the hits which satisfy appropriate E-value and coverage thresholds using single linkage clustering [20]. The result is the gene content matrix in which rows are the genomes and columns are the clusters and the values are the numbers of cluster members present in the given genome. The number of columns that have a non-zero entry in exactly *g *rows is the set of empirical data *γ_g _*used in the supergenome estimates in models B, C, D, and E.

Model F utilizes the number of gene acquisitions estimated by Count. The input to Count is the phylogenetic tree topology from the Microbes Online and the gene content matrix used for computing *γ_g_*. Because the gene content matrix is the empirical data set used for all models, robust supergenome estimates should agree at least qualitatively.

## Results

### Estimates using gene frequencies directly

**Figure 2:**
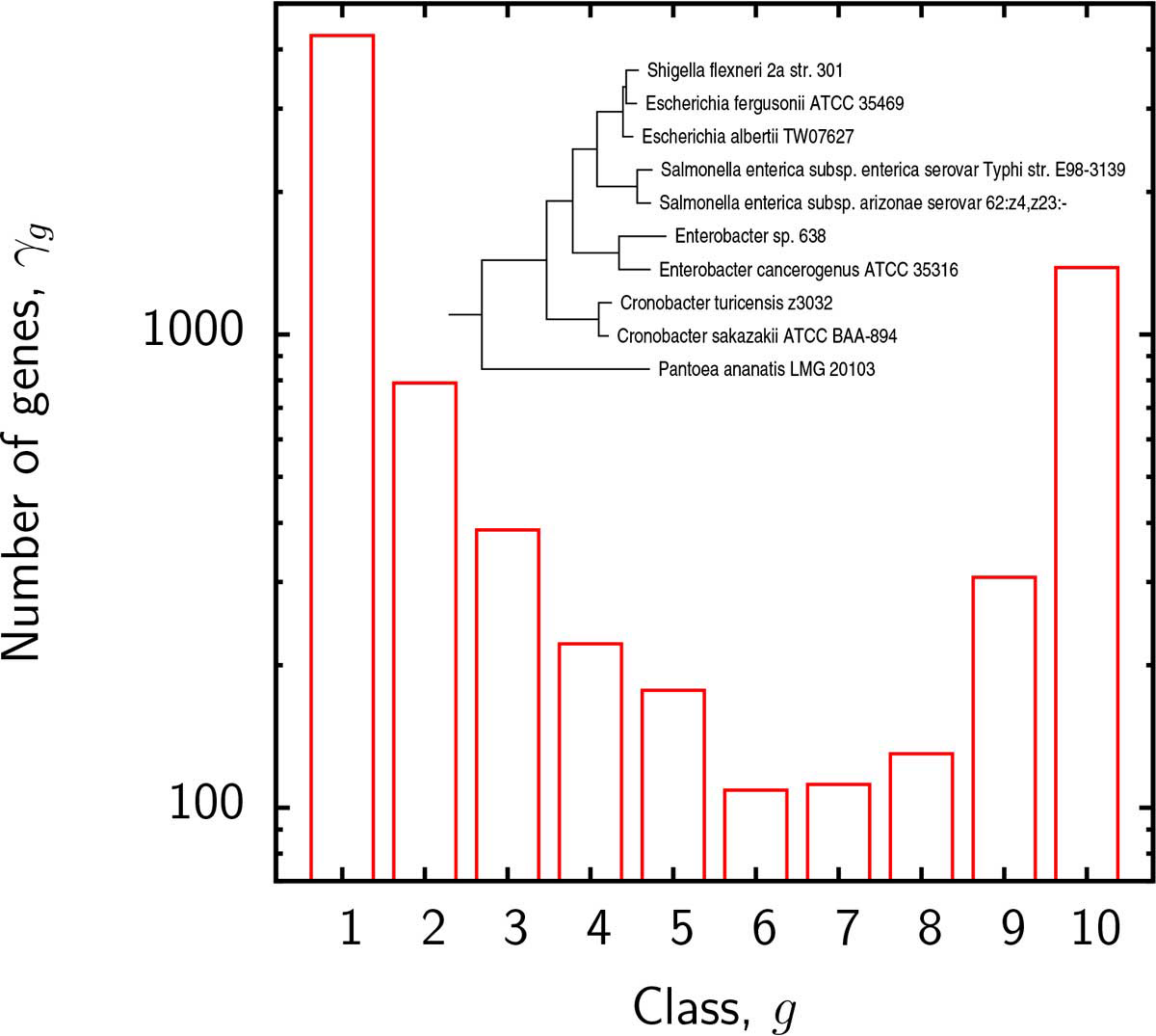
**Empirical numbers *γ_g_* of genes found in exactly *g *genomes in group 3**. Inset: the phylogenetic tree for group 3.

**Figure 3:**
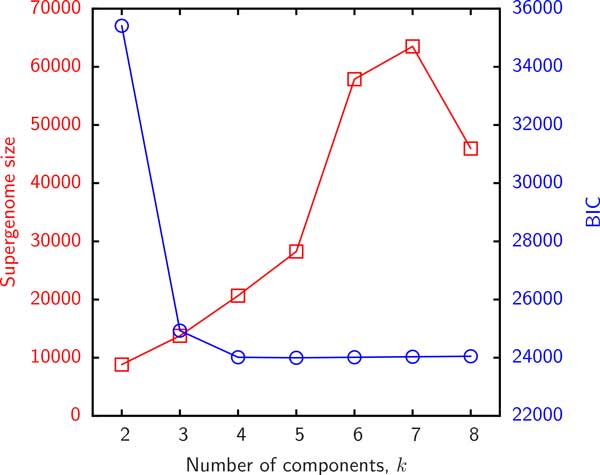
**The result of the binomial mixture model fit to the gene frequencies in Figure 2 as a function of the number of components**. Left (red) y-axis shows the estimated supergenome size and the right (blue) axis shows the BIC.

We first present the results of supergenome size estimates using the binomial mixture model (B). The essential parameter in this model is the number *k *of distinct sub-reservoirs. It is instructive to examine the variation of the *BIC *and the estimated supergenome size with *k*. Figure 2 displays the empirical gene frequencies *γ_g _*for group 3 and Figure 3 shows the *BIC *and supergenome estimates for this group as a function of the number of binomial mixture components *k*. Supergenome estimates vary by almost an order of magnitude whereas the *BIC *curve is roughly flat for *k >*3. We set *k *= 3 (i.e. 2*k − *2 = 4 adjustable parameters) for the remainder of this analysis to compare the results to those obtained with the other models which include 2 or 3 adjustable parameters.

Columns 5 and 6 of Table 1 show the results of the binomial mixture fits with *k *= 3 components to the empirical gene frequencies *γ_g_*, and column 7 shows the capture/recapture (model C) estimate of the supergenome size *S*. Model D uses essentially the same method of fitting the empirical gene frequencies as the binomial mixtures model B but instead of assuming that genes originate from several distinct homogeneous reservoirs, the gain probabilities in the reservoir are taken to be Gamma distributed.

**Figure 4:**
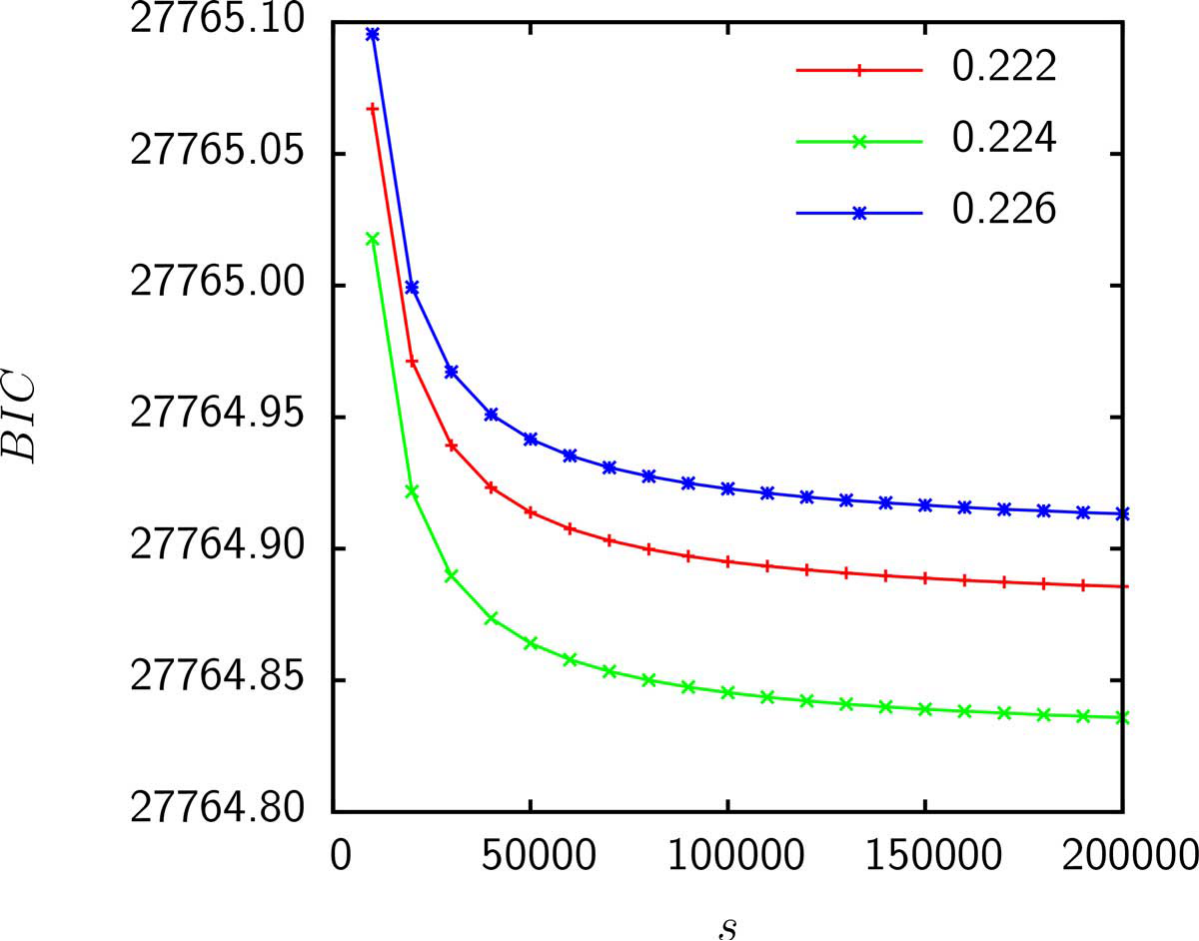
**The *BIC *for the fit of model D to the gene frequencies of group 3 as a function of *s***. The product *αs *was held constant and is shown in the legend.

Model D has 2 parameters: the shape parameter *α *of the Gamma distribution and the relative supergenome size *s *= *S/M *where *M *is the genome size. Figure 4 shows that the likelihood is maximized (or, equivalently, the *BIC *is minimized) when *α *tends to 0 whereas *s *tends to infinity such that the product *αs *is fixed. Thus, if gain probabilities are Gamma distributed, empirical data are consistent with an infinite supergenome.

The minimum *BIC *of model D, shown in column 8 of Table 1, is generally greater than that for model B which indicates that several homogeneous reservoirs reflect the supergenome composition better than the Gamma distributed gain probabilities.

Model E is essentially model D propagated along the branches of a tree. Therefore, an additional parameter, the loss rate *R*, is introduced to reflect the intensity of the gene flow from the reservoir to the genomes. Genomes become independent random samples of the reservoir in the *R *→ *∞ *limit. Given *R *we simulate the gene flow from the reservoir to the genomes on the phylogenetic tree, and compute the expected probabilities *θ_g_*, the likelihood via Eq. (4) and the *BIC*. The *BIC *is then minimized with respect to *R, S *and *α*.

**Figure 5:**
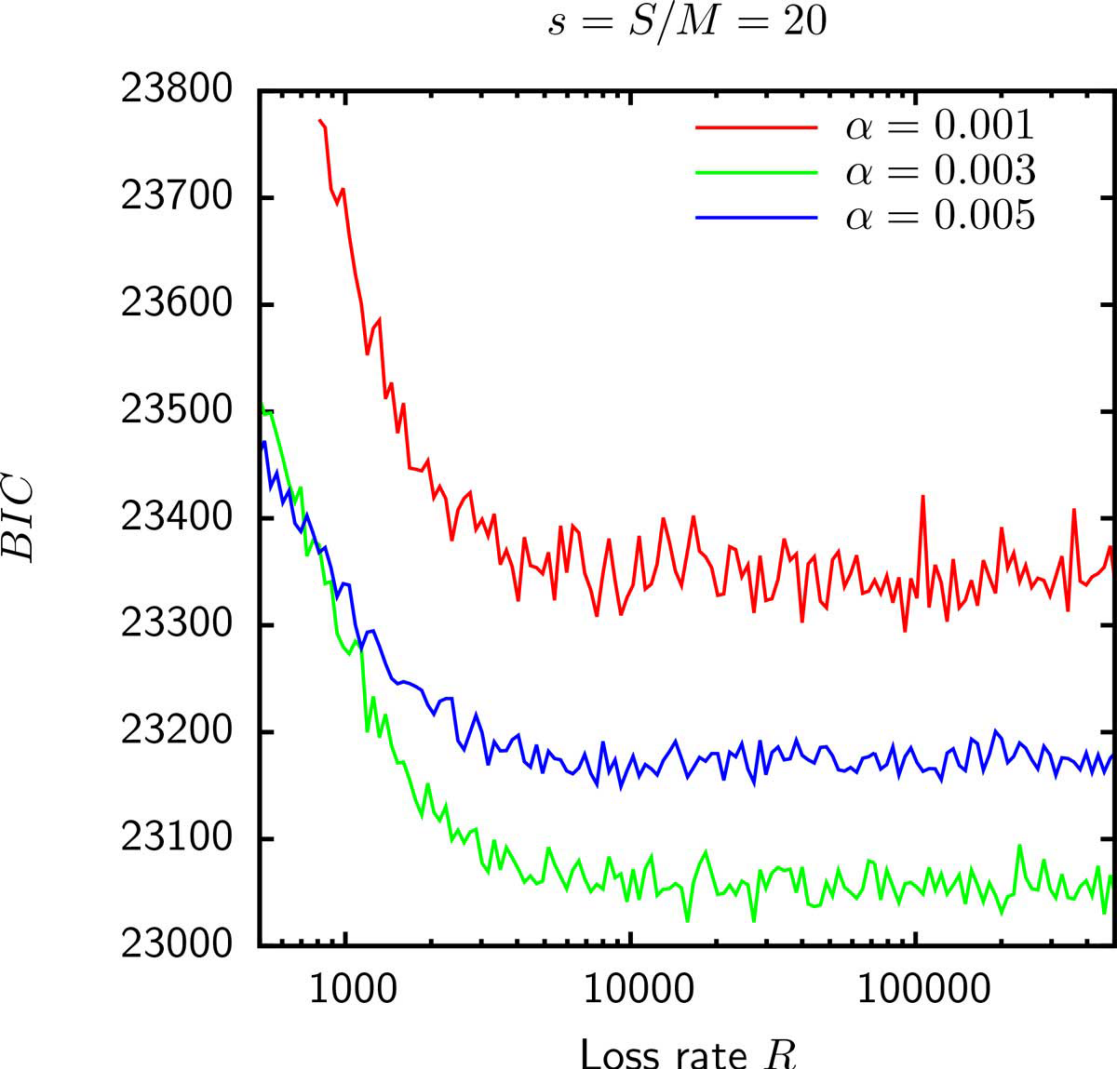
**The *BIC *for the model E fit to the gene frequencies in group 6 is minimized in the *R → ∞***. The same situation is observed in groups 1, 4, 5, and 8.

**Figure 6:**
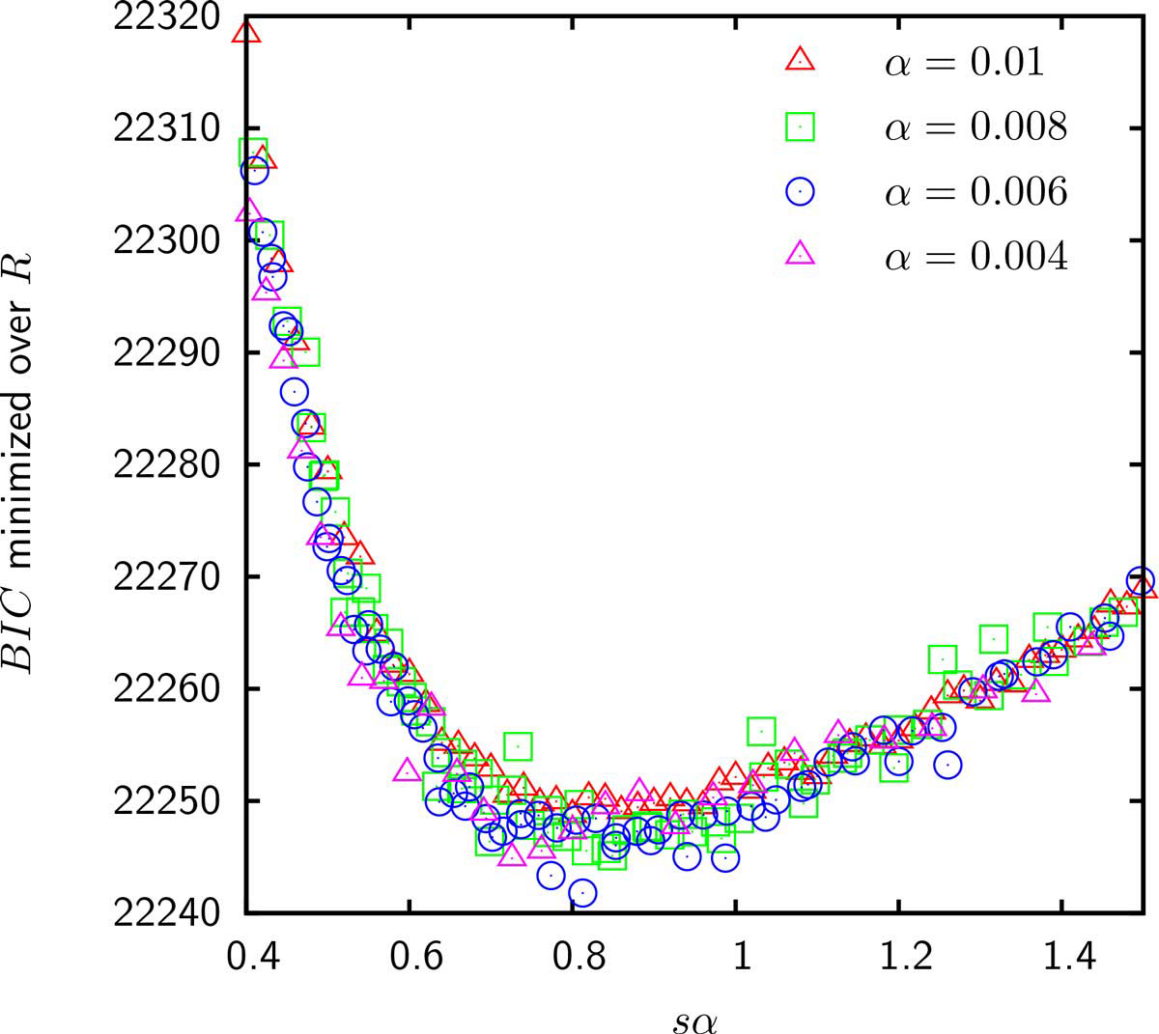
***BIC *minimized over the loss rate *R *for group 2 is a function of only the product of *α *and *s***. All other groups exhibit identical behavior of the *BIC *minimized over *R*.

As shown in Figure 5, the *BIC *for the model E fit to the gene frequencies in group 6 is minimized when *R *tends to infinity. This result indicates that for the Gamma distributed gain probabilities, the empirical gene frequencies in group 6 as well as groups 1, 4, 5 and 8 (data not shown) are consistent with being random independent samples of the reservoir. The best fit of the gene frequencies in groups 2, 3, 7 and 9 occurs at a finite *R *for which genomes retain a modest fraction of ancestral genes and hence cannot be considered independent. In addition, as shown in Table 1 when likelihood is maximized for a finite loss rate, model E is an improvement over model D. However, as shown in Figure 6 the supergenome size *S *still cannot be estimated because the *BIC *seems to be a function of only *sα *(where *s *= *S/M *is the relative supergenome size). Therefore, just as in model D, the goodness of fit does not change when *α *tends to 0 simultaneously with *s *tending to infinity so that the product *sα *is fixed.

### Estimates using reconstructed acquisitions

**Figure 7:**
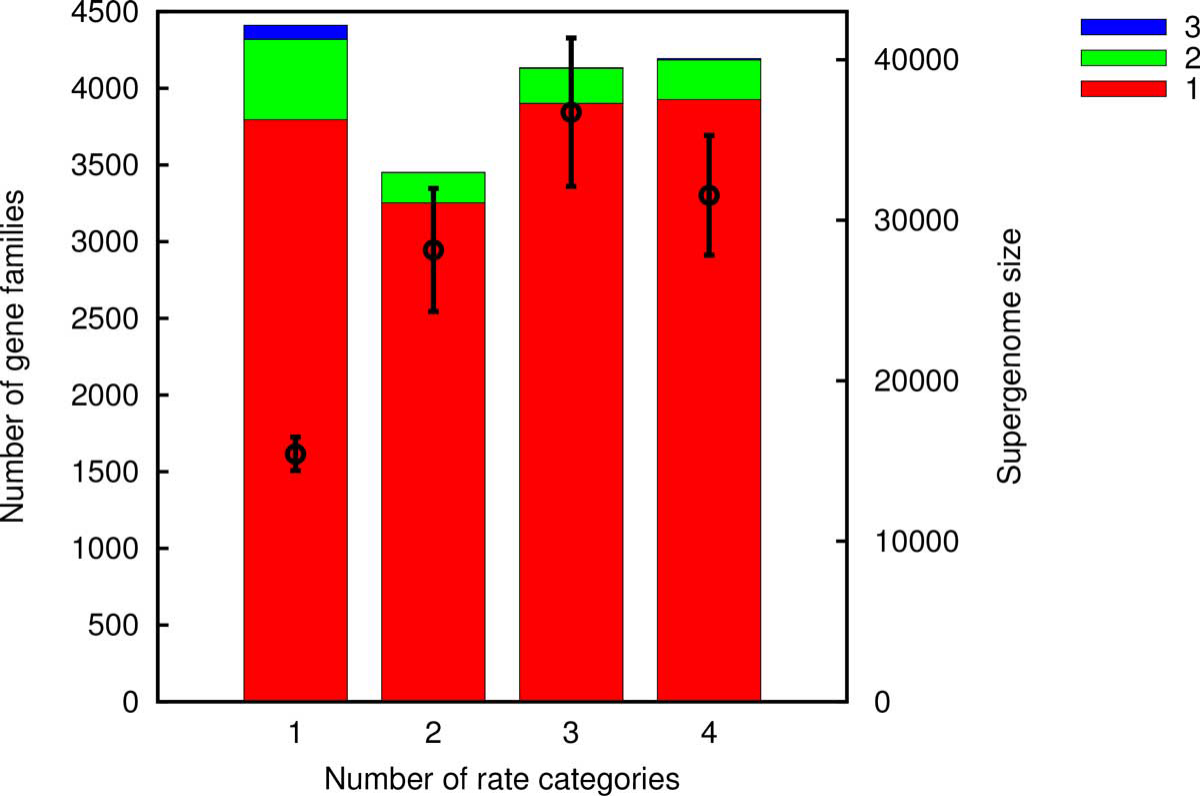
**Estimated number of gene families gained once, twice and thrice as a function of the Count model complexity for group 2**. The symbols with error bars (right y-axis) are the corresponding supergenome size estimates using the uniform gain probability Model F1.

Models F1 and F2 do not deal with gene frequencies directly but rather utilize the gene acquisitions reconstructed from the gene content of the extant genomes and the phylogeny using the ML reconstruction package Count. Count assumes that the dynamics of each gene family are described by a birth-death model with per-family rates of gain, loss and duplication. The rates are assumed to originate from several Gamma distributions. The number of categories of Gamma distributions, i.e. the model complexity, is the essential variable in the ancestral reconstruction process. The maximum likelihood scenario of losses and gains depends on the model complexity as shown in Figure 7. We selected a high complexity model with 4 Gamma distributed categories for loss, duplication and transfer rates yielding a total of 64 combinations. Parameter estimation in higher complexity models tends to be less reliable and our choice of 4 categories represents a compromise between accuracy and precision.

Table 2 summarizes the supergenome size estimates assuming that the gain probability is uniform (model F1). Whereas estimates using only gains (acquisitions of families not present in the genome) vary by two orders of magnitude, when both gains and expansions are assumed to be acquisitions, supergenome estimates are remarkably robust.

**Table 2:** Maximum likelihood estimates of the supergenome size using reconstructed gene gains (column 5) and gains plus expansions (column 4) using uniform gain probabilities (model F1).

Group	Gains *P *:*K*	gains+exp *P *:*K*	gains *S*	gains+exp *S*
1	1312:1445	1789:2028	7355 *± *1195	7910 *± *939
2	4193:4468	4805:5600	34783 *± *4019	17805 *± *1137
3	6227:5647	6956:8003	64763 *± *7001	27853 *± *1564
4	4577:4777	5346:5923	55434 *± *7618	28387 *± *2205
5	3762:3844	4744:5239	88790 *± *19330	25944 *± *2180
6	3805:3816	4651:5071	660500 *± *397000	28893 *± *2659
7	5551:5947	6462:7351	42643 *± *4091	27885 *± *1713
8	4920:5198	5873:6792	46838 *± *5414	22776 *± *1360
9	4730:5020	5745:6483	41751 *±*4711	26266 *±*1781

**Figure 8:**
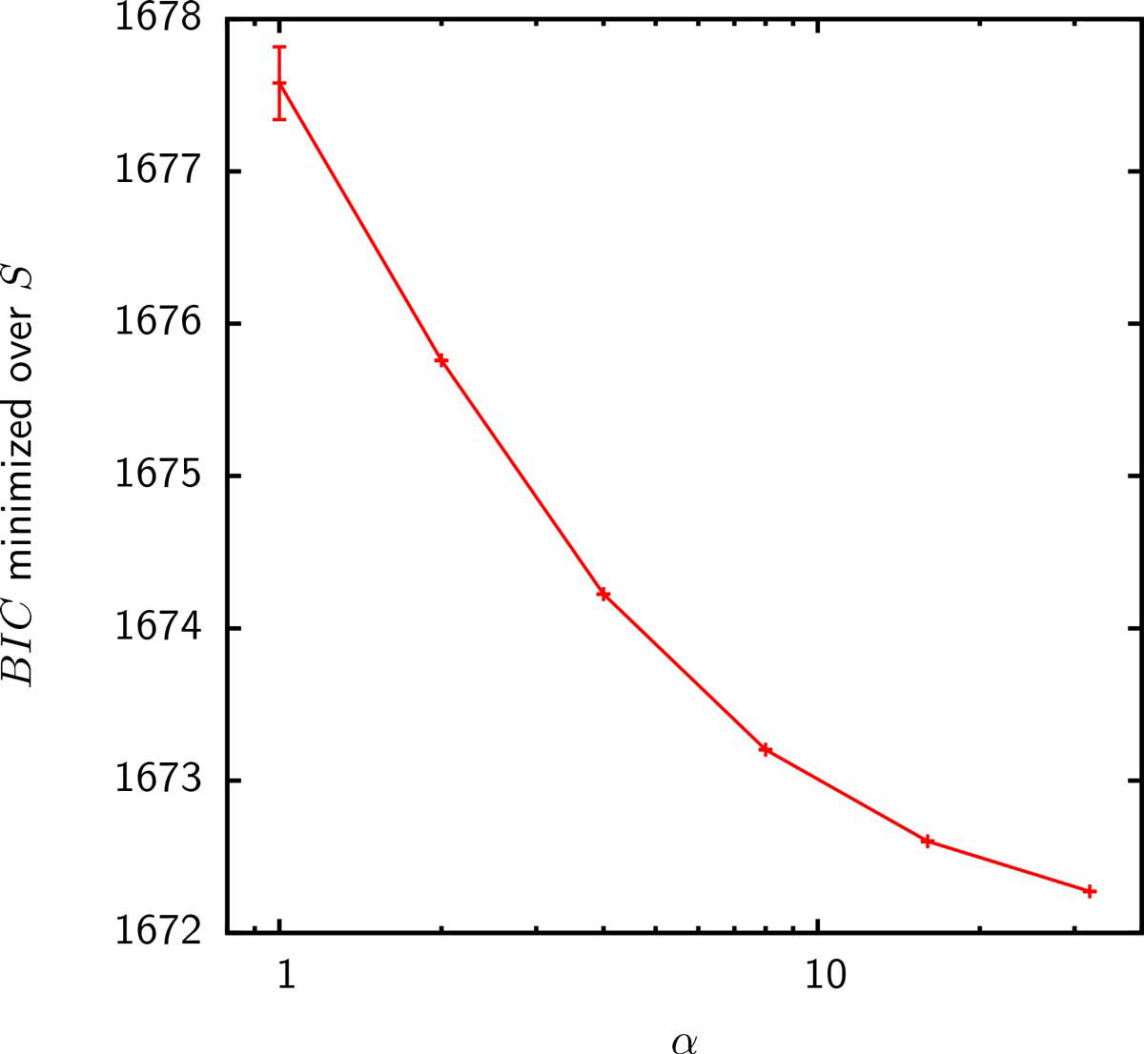
***BIC *minimized over *S *as a function of *α *for the fit of model F2 to the inferred gene acquisitions discounting expansions in group 4**. The *α → ∞ *limit of model F2 is the uniform gain probability model F1. The minimum BIC for the fits of model F2 to the acquisitions inferred in the rest of the groups behaves in a similar fashion.

To compare models F1 and F2, we compute the *BIC *using the likelihood in Eq. (10). When only gains are counted as acquisitions, the Gamma distributed gain probability did not result in an improved fit and the maximum likelihood (minimum *BIC*) was achieved in the *α → ∞ *limit for all groups (Figure 8). Because the Gamma distribution becomes sharply peaked, model F2 reduces to the uniform gain probability model F1 in the *α → ∞ *limit. Thus, the homogeneous reservoir hypothesis seems to be more consistent with the pattern of gene gains inferred by Count when only gains are considered to be acquisitions from the supergenome.

A different picture emerges when both gains and expansions are considered to be acquisitions from the supergenome. As shown in Table 3 the fit of the Gamma distributed gain probability model F2 to the inferred gains is an improvement over the uniform gain probability model F1 for all groups with the exception of group 7. However, in some cases, the best fit occurs in the *α → *0. Consequently, as in models D and E, the estimated supergenome size diverges.

**Table 3:** Comparison between supergenome estimates using uniform gain probabilities (model F1) and Gamma distributed gain probabilities (model F2).

Group	F1: *S*	F1: *BIC*	F2: *BIC*	F2: *α*	F2: *S*
1	7910	1481	1455	0.00	*∞*
2	17805	4637	4590	0.36	59000
3	27853	6336	6191	0.00	*∞*
4	28387	3807	3800	0.85	55700
5	25944	3332	3275	0.00	*∞*
6	28893	2912	2911	1.42	43900
7	27885	5423	5427	*∞*	28900
8	22776	5453	5393	0.14	140000
9	26266	4618	4611	2.9	31000

Gains and expansions are counted as gene acquisitions.

## Discussion

Here we assumed that genomes evolve by randomly acquiring genes from a reservoir we denote supergenome. We explored several methods of estimating the size and composition of this supergenome. The methods are based on combinations of 2 assumptions: a) genomes are assumed to be random independent samples from the reservoir or, alternatively, assumed to have evolved from a common ancestor by a stochastic process of gene replacement; and b) the reservoir is assumed to consist of one or more homogeneous sub-reservoirs or the gene gain probability is assumed to be Gamma distributed. The models were fit to two kinds of empirical data: a) gene frequencies *γ_g _*defined as the number of gene families found in *g *out of *G *genomes, and b) the number *n_k _*of families that have been acquired *k *times as estimated by the ancestral reconstruction package Count.

The data set used to explore the robustness of supergenome estimates consisted of 9 groups of 10 *Enterobacteriacae *with similar genome sizes that shared the same common ancestor. We found that the supergenome estimates using gene frequencies *γ_g _*were possible only when the supergenome was assumed to be composed of *k *homogeneous sub-reservoirs and even then the estimate varied by about an order of magnitude as a function of *k*. When the supergenome was assumed to consist of gene families with Gamma distributed gain probabilities, the empirical data were consistent with the supergenome of infinite size. In 5 groups the data were consistent with the genomes being random independent samples of the supergenome but the genomes in the remaining 4 groups were correlated and the best fit of the model with the evolution on a tree component occurred at a finite evolution rate *R*. Even then, a reliable estimate of the supergenome size could not be obtained because the likelihood of the data seem to depend only on the product of the Gamma distribution's shape parameter *α *and the relative supergenome size *s*.

Supergenome estimates based on the Count-estimated number of gene gains could be computed reliably but only when the probability of gaining any gene from the supergenome was the same. The estimates were moderately sensitive to the complexity of the underlying model used for the reconstruction of the gains. The estimates of the supergenome size obtained using the binomial mixtures model did not correlate with those obtained using reconstructed multiple gains (Spearman rank-correlation *P*-value of 0.46).

To summarize, supergenome size estimates using simple models of genome evolution and extant gene frequencies did not appear robust. If the gain probabilities were assumed to be Gamma distributed, the gene frequencies seemed to suggest infinite (open) supergenomes. Under the assumption of several homogeneous subreservoirs, finite supergenome estimates were obtained but the size of the estimated supergenomes strongly depended on the number of reservoirs. In contrast, when the the likelihood was computed using reconstructed numbers of gains, more reliable, finite estimates of the supergenome size were obtained. The lower bound on the supergenome size, obtained by including both gains of absent families and expansions of existing families, was found to be substantially less variable than the upper bound obtained by excluding expansions. For the majority of the analyzed groups of bacteria, the estimated supergenomes were roughly an order of magnitude larger than the mean genome size. When only gains of absent families were considered to be acquisitions from the supergenome, the homogeneous finite supergenome model was more consistent with the data than the Gamma distributed gain probability model. The value of the supergenome size was only moderately sensitive to the complexity of the birth-death model used for the ancestral reconstruction. When expansions were considered to be acquisitions, the model with the Gamma distributed gain probabilities was more consistent with the data. In this case, however, the supergenome estimates could not be reliably obtained in all groups.

In summary, the models with the Gamma distributed gain probabilities yielded highly variable and inconsistent supergenome size estimates, suggesting that this distribution does not reflect the process of gene acquisition by prokaryotes. In the same vein, none of the evolutionary models explored here yielded robust supergenome estimates, suggesting that the complexity of these models is inadequate to mimic the process of genome evolution. In contrast, the more empirically-rooted approach that used reconstructed numbers of gene gains for the supergenome estimation yielded more consistent values and seemed to be converging on about an order of magnitude difference between the sizes of a supergenome and a typical genome in the respective group for the majority of bacteria.

## Competing interests

The authors declare that they have no competing interests.

## Authors' contributions

AEL, YIW and EVK designed the model, AEL performed numerical simulations and analyzed the results, AEL, YIW and EVK wrote the manuscript.

## Supplementary Material

Additional file 1 Click here for file

## Declarations

Publication costs for this article are funded by the intramural funds of the Department of Health and Human Services, Institutes of Health, National Library of Medicine.

This article has been published as part of *BMC Genomics *Volume 15 Supplement 6, 2014: Proceedings of the Twelfth Annual Research in Computational Molecular Biology (RECOMB) Satellite Workshop on Comparative Genomics. The full contents of the supplement are available online at http://www.biomedcentral.com/bmcgenomics/supplements/15/S6.
