# Supplementary figures and images for: Estimation of prokaryotic supergenome size and composition from gene frequency distributions
Source: BMC Genomics. 2014 Oct 17;15(Suppl 6):S14. doi: 10.1186/1471-2164-15-S6-S14 (PMC4240607; doi:10.1186/1471-2164-15-S6-S14)

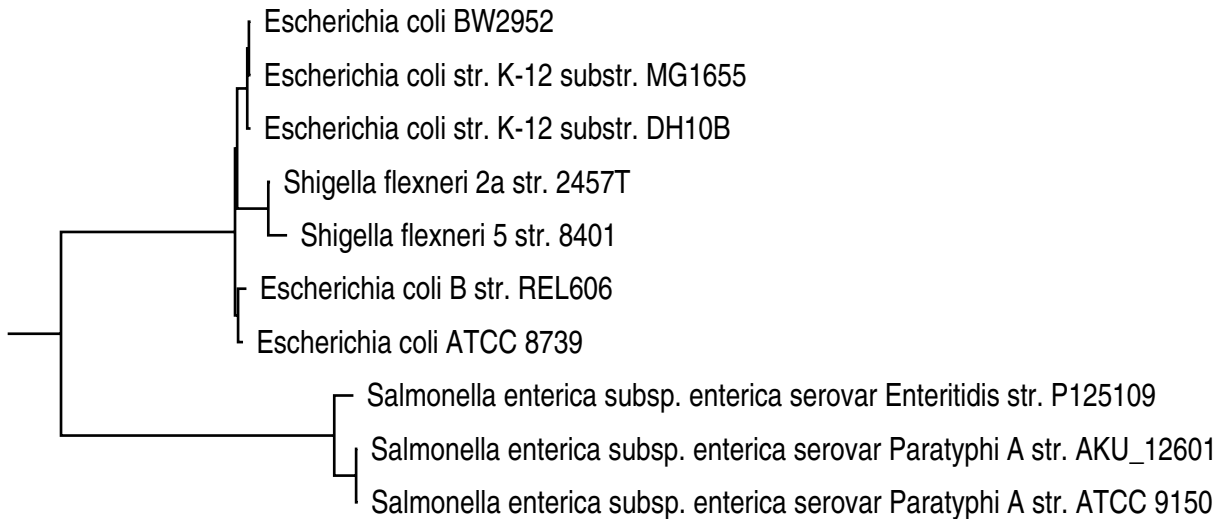

Supplement: Additional file 1 [file 1471-2164-15-S6-S14-S1.zip › group1.pdf]

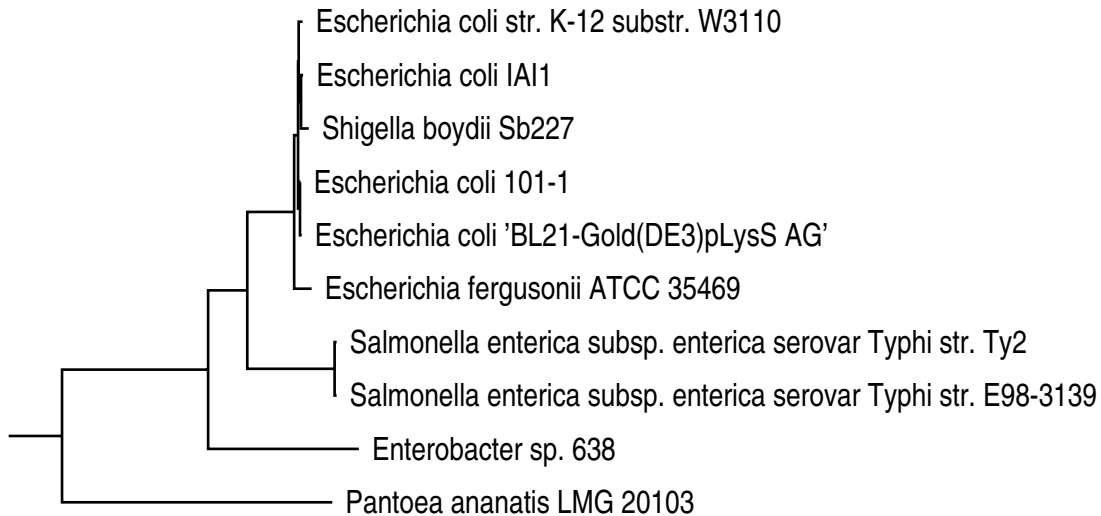

Supplement: Additional file 1 [file 1471-2164-15-S6-S14-S1.zip › group2.pdf]

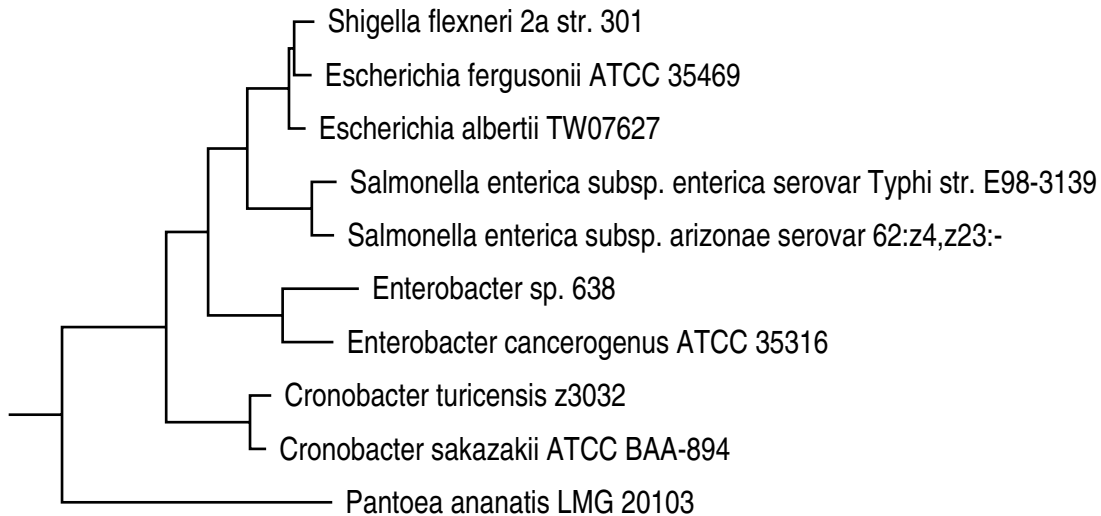

Supplement: Additional file 1 [file 1471-2164-15-S6-S14-S1.zip › group3.pdf]

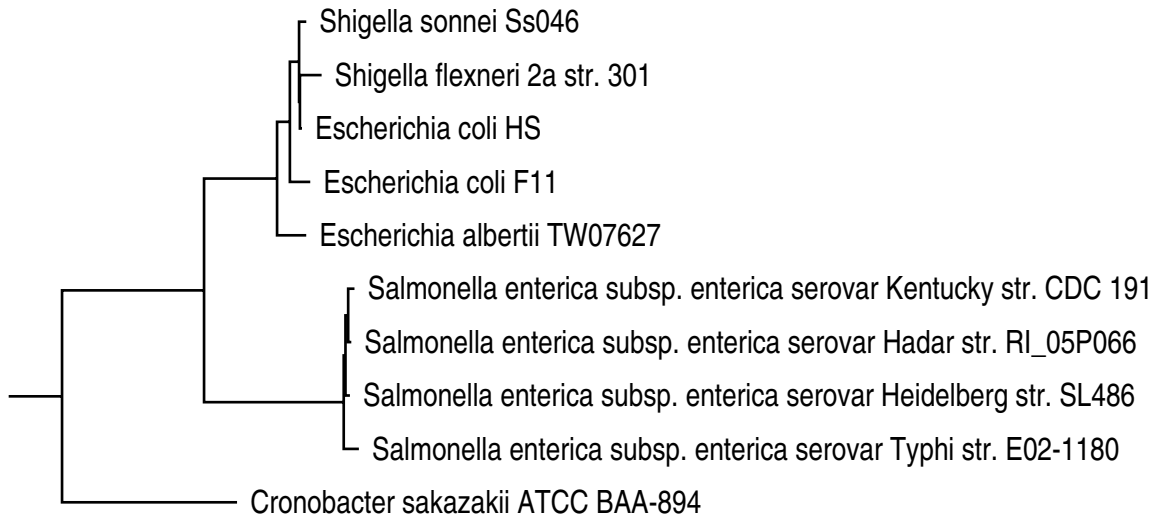

Supplement: Additional file 1 [file 1471-2164-15-S6-S14-S1.zip › group4.pdf]

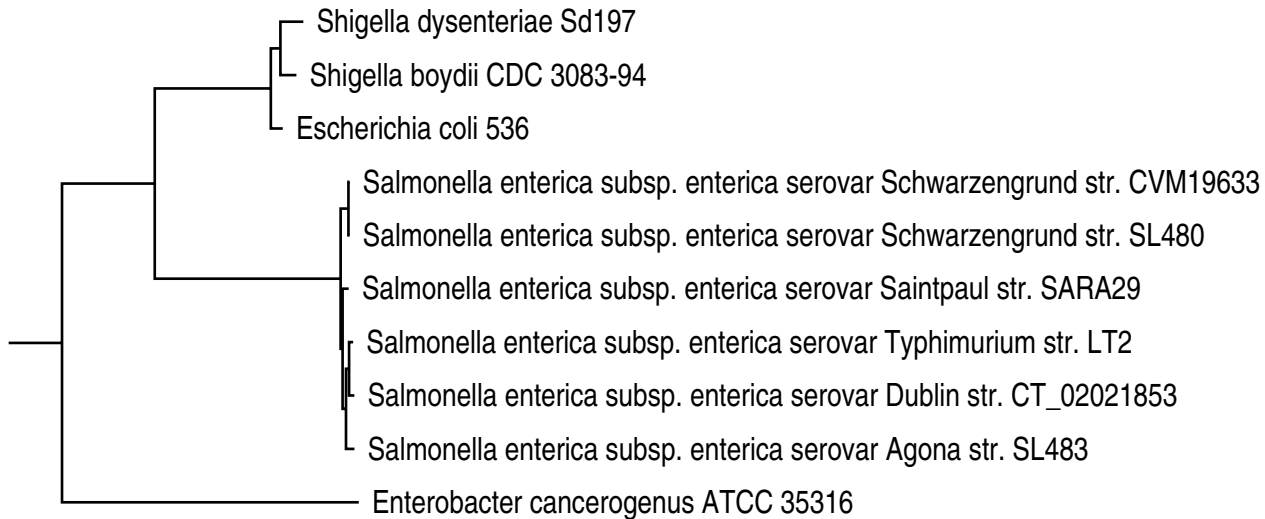

Supplement: Additional file 1 [file 1471-2164-15-S6-S14-S1.zip › group5.pdf]

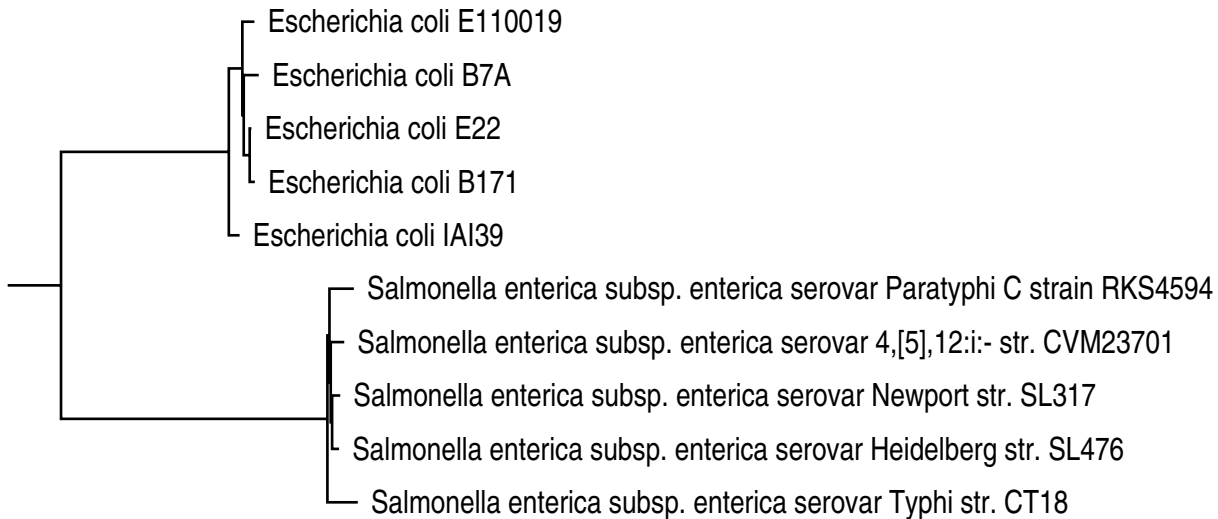

Supplement: Additional file 1 [file 1471-2164-15-S6-S14-S1.zip › group6.pdf]

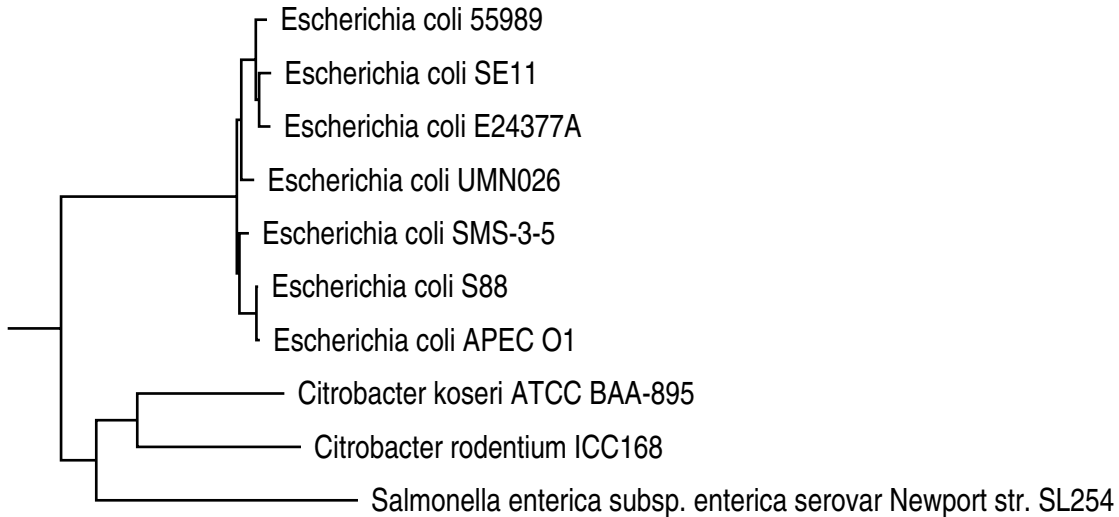

Supplement: Additional file 1 [file 1471-2164-15-S6-S14-S1.zip › group7.pdf]

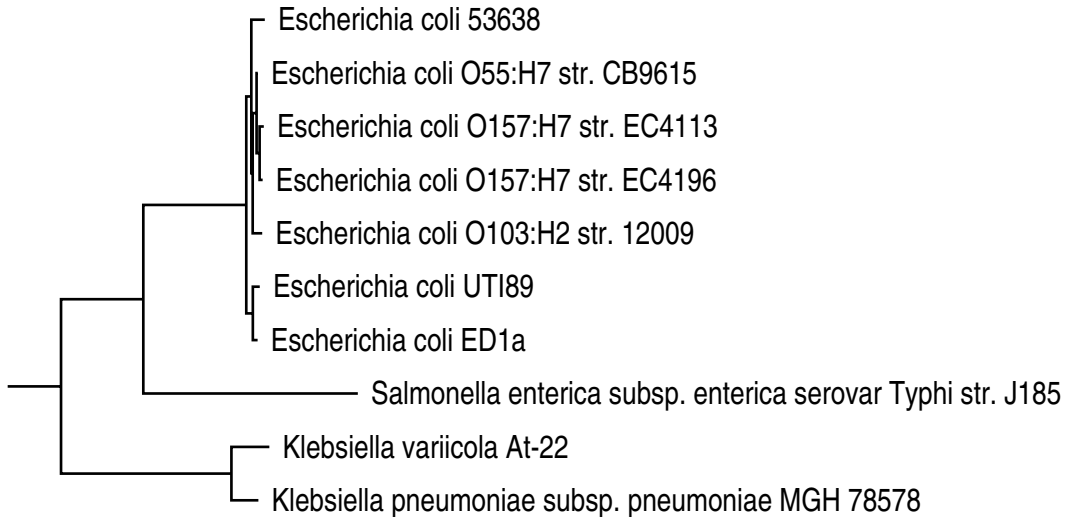

Supplement: Additional file 1 [file 1471-2164-15-S6-S14-S1.zip › group8.pdf]

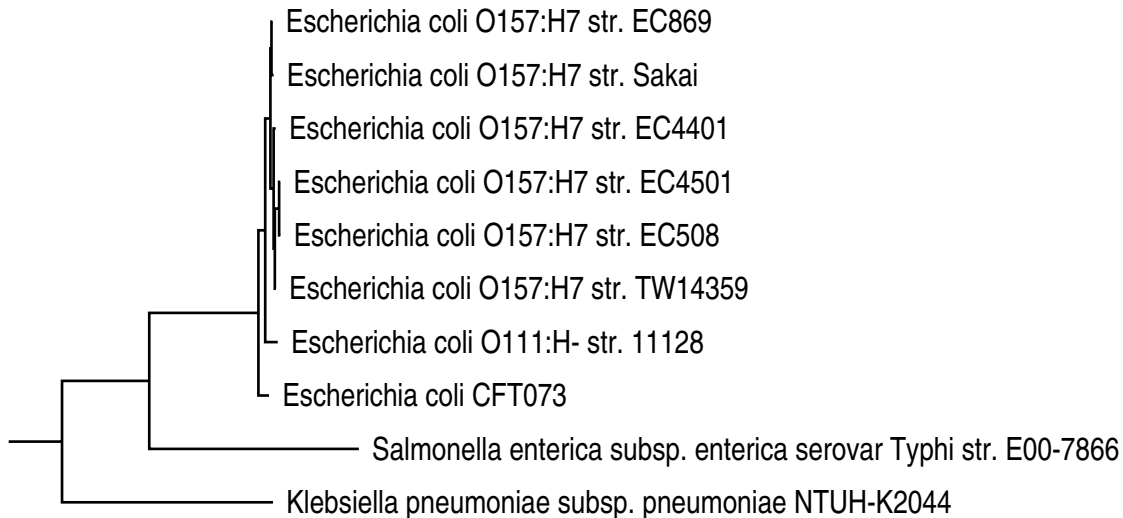

Supplement: Additional file 1 [file 1471-2164-15-S6-S14-S1.zip › group9.pdf]
